# Multi-Modal Multi-Array Electrochemical and Optical Sensor Suite for a Biological CubeSat Payload

**DOI:** 10.3390/s24010265

**Published:** 2024-01-02

**Authors:** Saeyoung Kim, Sanghyun Park, James Jungho Pak

**Affiliations:** School of Electrical Engineering, Korea University, Seoul 02841, Republic of Korea; macx93@korea.ac.kr (S.K.); yh99gosh22@korea.ac.kr (S.P.)

**Keywords:** multi-modal, multi-array, electrochemical, electrode, optical, detector, CubeSat, payload

## Abstract

CubeSats have emerged as cost-effective platforms for biological research in low Earth orbit (LEO). However, they have traditionally been limited to optical absorbance sensors for studying microbial growth. This work has made improvements to the sensing capabilities of these small satellites by incorporating electrochemical ion-selective pH and pNa sensors with optical absorbance sensors to enrich biological experimentation and greatly expand the capabilities of these payloads. We have designed, built, and tested a multi-modal multi-array electrochemical-optical sensor module and its ancillary systems, including a fluidic card and an on-board payload computer with custom firmware. Laboratory tests showed that the module could endure high flow rates (1 mL/min) without leakage, and the 27-well, 81-electrode sensor card accurately detected pH (71.0 mV/pH), sodium ion concentration (75.2 mV/pNa), and absorbance (0.067 AU), with the sensors demonstrating precise linear responses (R^2^ ≈ 0.99) in various test solutions. The successful development and integration of this technology conclude that CubeSat bio-payloads are now poised for more complex and detailed investigations of biological phenomena in space, marking a significant enhancement of small-satellite research capabilities.

## 1. Introduction

### 1.1. Background

**Figure 1:**
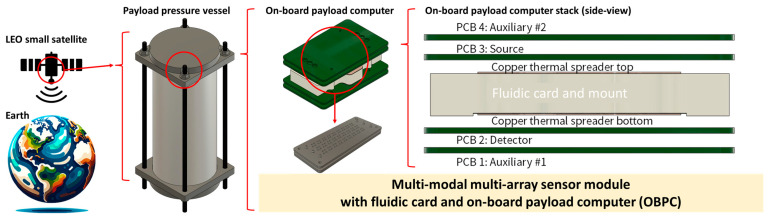
Conceptual diagram of a small satellite in LEO with an on-board payload computer and a fluidic card capable of multi-modal multi-array electrochemical and optical sensing for conducting biological experiments in space.

CubeSats, recognized as a specific category of small satellites consisting of multiple cubic units each measuring 10 cm × 10 cm × 10 cm and typically weighing less than 2 kg [1], have been acknowledged as both capable and more attainable means for executing scientific endeavors in space [2,3,4,5,6,7,8]. The scope of this paper was narrowed to concentrate on CubeSats utilized as orbital laboratories, particularly for the examination of microorganisms in extraterrestrial environments. These microorganisms are deemed vital for the development of life-support systems in prospective manned deep-space expeditions [9,10,11]. A schematic representation of such an autonomous orbital laboratory, situated in low Earth orbit (LEO), tasked with the cultivation and analysis of microbial cells to discern the biological impacts of microgravity and cosmic radiation, is depicted in Figure 1.

In CubeSat missions, sensors are vital for data acquisition and monitoring, tailored to fit the platform’s limited resources. Optical sensors enable Earth and space observations, aiding environmental and celestial research [12]. Radiation sensors provide data on cosmic and solar rays, essential for space weather studies and astronaut safety. Sensors that analyze the impact of space on microorganisms are also crucial, but quite lacking in capability now as their limitations will be explained subsequently. However, they are important for advancing astrobiology and life-support technology for space travel. Despite their size, CubeSats’ integrated, miniaturized sensors are crucial for diverse scientific explorations, enhancing our knowledge of space.

### 1.2. Prior Research and Limitations

Table 1 catalogs the target organisms and measured parameters for a series of past biological experiments in orbit, enabled by small satellite payloads including GeneSat-1 [13,14,15], PharmaSat 1 [16,17,18], O/OREOS [19,20,21,22], EcAMSat [23,24,25], and Biosentinel [26,27,28,29,30]. These satellites predominantly featured optical absorbance sensors to track microbial growth. In contrast, SporeSat [31], which launched in 2014, deviated from this norm by incorporating Ca^2+^ ion-sensitive electrodes, omitting optical absorbance sensors entirely. Reports on the Gravisat [32] mission suggest the inclusion of electrochemical ion-sensitive electrodes; however, comprehensive details on its design, fabrication, or functional testing remain unpublished. Notably, each satellite documented with precision to date has been equipped exclusively with one type of sensor—either optical or electrochemical—without integrating both sensing capabilities in a single platform.

The exclusion of electrochemical ion sensors from these autonomous orbital laboratories results in a significant analytical deficit. Such an omission precludes the quantification of proton concentration, a critical determinant of the biochemical milieu that influences microbial metabolic pathways and cellular homeostasis [34]. The lack of pNa sensors further compromises the ability to monitor sodium ion dynamics, essential for elucidating cellular osmoregulation mechanisms and ion-driven transport across membranes, fundamental in astrobiological studies [35]. Additionally, the absence of electrochemical sensing impedes the real-time tracking of metabolic exchanges, such as electron transport chain fluxes or ionic translocations, which are pivotal in deciphering the adaptive responses of microorganisms to extraterrestrial environments [36]. This limitation not only narrows the scope of metabolic activity surveillance but also restricts the capacity to investigate the microorganisms’ environmental interactions, thereby curtailing the comprehensive understanding required to inform the design of closed-loop life-support systems and to advance the field of space biotechnology.

A CubeSat sensor system measuring pH, pNa, and absorbance, compared to one measuring only absorbance, offers considerable advantages for monitoring yeast growth. The ability to measure pH and pNa enhances growth condition monitoring, crucial for optimizing conditions and identifying stress responses, as yeast growth depends on both nutrient availability and the medium’s pH and ionic conditions. This system also provides deeper insights into metabolic activities, tracking changes in pH and ion concentrations, like acid production during fermentation [37]. It allows the earlier detection of contamination or anomalies, as pH and sodium ion fluctuations indicate such issues more promptly than absorbance alone [38,39]. In microgravity environments like those in CubeSats, these detailed data are essential for understanding yeast behavior in space, vital for long-term space missions. The system’s adaptability to different research needs, accommodating various yeast strains and experimental setups, and its ability to offer a comprehensive view of the yeast growth environment, enhance the overall quality and applicability of research data.

### 1.3. Main Purpose and Scope of This Work

This research specifically aimed to develop and validate a benchtop development model (DM) for an integrated sensor module, combining both electrochemical and optical sensors, designed for use in a 2U CubeSat payload. This model is particularly intended for application in future biological missions using CubeSats. The scope of our work involved comprehensive documentation and meticulous processes in several key areas: first, the design and fabrication of a fluidic card; second, the creation of a multi-array system of electrochemical electrodes; and third, the development and integration of hardware and firmware for the on-board payload computer (OBPC). The culmination of this research was a series of functional tests, conducted using a variety of liquid samples, to rigorously assess and confirm the operational readiness of the entire module for potential future space applications.

## 2. Materials and Methods

### 2.1. Fluidic Card

#### 2.1.1. Design

The design of the fluidic card is aimed at providing a designated space for the growth and maintenance of microbial cells. It consists of two polymer layers and the multi-modal multi-array electrode layer, forming a structure referred to as a ‘fluidic card’. This card encompasses numerous wells, which are used to house the microbial cells. Additionally, it features fluidic inlets and outlets that facilitate the circulation of growth media into and out of these wells, ensuring a continuous supply of fresh growth medium. An essential function of the fluidic card is to enable gas exchange between the wells and the external environment, while simultaneously containing the microbial cells and liquid growth media within its confines, thereby preventing any leakage.

**Figure 2:**
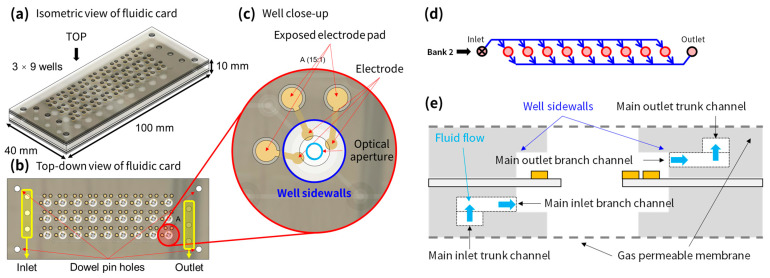
Three-dimensional CAD model of fluidic card and mount. (**a**) Isometric view of fluidic card. (**b**) Top-down view of fluidic card. (**c**) Single-well close-up from top-down view. (**d**) Conceptual flow diagram of fluidic card of a single bank from inlet to outlet. (**e**) Cross-sectional view of a single well.

Figure 2a illustrates the isometric view of the fluidic card, while Figure 2b presents the top-down perspective. This card is structured with 27 wells, arranged in 9 columns across 3 rows. These rows are sequentially designated as bank #1, bank #2, and bank #3, starting from the top. In each row, the 9 wells are interconnected through fluidic channels. The card incorporates 3 inlets and 3 outlets, with each pair dedicated to a single bank. The dimensions of the fluidic card are 100 × 40 × 10 mm^3^. The wells themselves are each 4 mm in diameter and have a depth of 5 mm, leading to the bottom of a gas-permeable membrane. This configuration yields a liquid volume of 62.8 mm^3^ per well. Additionally, each well features several holes for the insertion of electrical pogo-pins. These pins, extending from the top PCB, establish contact with the fluidic card’s electrode layer. While the number and arrangement of these holes can vary based on the setup, the current design includes three holes per well: one for the pH working electrode (WE), one for the pNa WE, and one for the shared Ag/AgCl reference electrode (RE). A close-up view of a single well with its 3 electrodes is shown in Figure 2c.

A simplified diagram of the flow path for the fluidic card is shown in Figure 2d along with a cross-sectional view of a single well in Figure 2e. The liquid growth medium enters the inlet from the bottom left of the figure, fills the well from the bottom-up, and exits out of the outlet at the top right of the figure. The inlets and outlets are plugged with a liquid-permeable (but solid-filtering) membrane, while the bottom and top of the well are capped with a gas-permeable membrane. This allows the well to hold liquid whilst allowing gas exchange to occur.

#### 2.1.2. Fabrication and Assembly

Autodesk’s Fusion360 was used to 3D-CAD-model the fluidic cards. The core layers were 3D-printed in HT-90 resin using a high-resolution industrial 3D printer (ProJet MJP 2500/2500 Plus, 3D Systems, Rock Hill, SC, USA). Between each layer, pressure sensitive adhesive (Adhesive 300LSE, 3M, Maplewood, MN, USA) was used to stick all layers together. For the gas-permeable membrane, BreatheEasy^®^ sealing membranes (Z380059-1PAK, Merck, NJ, USA) were used. The fluidic card mounts were 3D-printed with ABS using Cubicon Single Plus (3DP-310F, HyVISION SYSTEM Inc., Seongnam-si, Republic of Korea). Silicon tubing of 2 mm inner diameter was used. A syringe pump (Standard Infusion Only Pump 11 Elite Syringe Pump, Harvard Apparatus, Holliston, MN, USA) and deionized (DI) water with or without red food coloring (JUNGWOO IND.CO, Daegu, Republic of Korea) were used for leakage and flow tests. Photographs and video were taken with an iPhone 11 (Apple, Cupertino, CA, USA).

#### 2.1.3. Flow and Leakage Test Process

Several quantitative and qualitative tests were conducted to evaluate the function of the fluidic card. There were three main evaluation criteria: (1) leakage, (2) critical flow-rate (before leakage or card rupture), and (3) well fill.

1. Criterion 1: Leakage
  a. Intra-bank leakage: liquid leakage from one bank well into other features such as pogo-pin holes or other wells within the same bank.
  b. Inter-bank leakage: liquid from one bank leaking into another bank.
  c. Overall leakage: liquid within the fluidic card well and channels leaking out of the card from the sides.
2. Criterion 2: Critical flow-rate
  a. The highest flow-rate a system can handle before experiencing rupture or leakage.
3. Criterion 3: Well fill
  a. Whether the wells fill completely with liquid is crucial, as trapped air bubbles can prevent the electrochemical sensor electrodes from making proper contact with the test liquid.

The leakage (Criterion 1) of the fluidic card was tested by using a syringe pump. A 10 mL syringe was installed within the syringe pump and connected to the inlet of the fluidic card by a 2 mm inner diameter silicone tube. Infusion rates (i.e., flow-rate) were tested at 5, 20, 100, 200, and 1000 μL/min. Leakage was evaluated with visual inspection. The critical flow-rate (Criterion 2) of the fluidic card was tested by varying the flow-rate setting within the syringe pump. The critical flow-rate was set to the highest flow-rate value of the syringe pump without leakage. The well fill (Criterion 3) was tested through visual observation of the wells.

### 2.2. Multi-Modal Multi-Array Electrochemical Electrode and Optical Sensor Module

#### 2.2.1. Design

**Figure 3:**
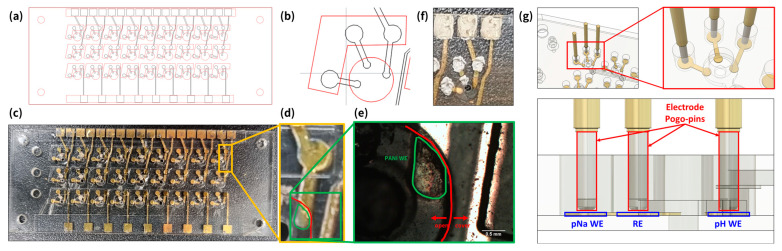
Multi-modal multi-array electrode design and fabrication process. (**a**) AutoCAD design files (black line for electrode mask design and red line for passivation layer). (**b**) Close-up of a single-well with its accompanying 3 electrodes (from left to right: pNa WE, shared RE, and pH WE). (**c**) Photograph of pH and pNa sensor electrode array after Cr and Au deposition. (**d**) Close-up of a single pH WE before electropolymerization. (**e**) Close-up photograph of a PANI-electropolymerized pH WE. (**f**) Close-up photograph of a single-well and its 3 electrodes with Ag ink layered on top for protection. (**g**) Three-dimensional CAD model of pogo-pins making contact with the pogo-pin pads of the electrode layer.

Here, we detail the design of the 27-well multi-modal multi-array sensor capable of measuring the pH, pNa, and absorbance of each well. The electrochemical electrode component of the multi-modal multi-array sensor is sandwiched between the top and bottom layers of the fluidic card and is integrated into the payload computer between the detector and source PCB. Figure 3 shows the design files and photograph of the electrochemical electrode portion of the multi-modal multi-array sensor. Figure 3a,b show the technical drawings used for electrode fabrication. Figure 3c,d are photographs of the electrodes after Cr and Au deposition but before WE functionalization and pad protection. Figure 3e,f are close-up pictures of the electrodes after pH WE polyaniline (PANI) functionalization [40,41], pNa WE sodium ionophore functionalization [42,43], and Ag ink pad protection.

To establish the 81 necessary electrical connections with the source PCB, which is located above the electrode layer, the use of vertical, spring-loaded pogo-pins was determined to be an effective solution. Each electrode, with a diameter of approximately 500 μm, is connected to a pogo-pin pad, which has a diameter of 1 mm. This setup is complemented by a 1 mm diameter pogo-pin head, soldered to the source PCB, that exerts pressure onto the pogo-pin pad, as depicted in Figure 3g. To safeguard these pogo-pin pads against potential damage from the pressure exerted by the pogo-pins, they were coated with a fine layer of Ag ink.

The multi-array sensor’s main role is to track pH, pNa, and absorbance levels in the microorganisms present in each well. It achieves this by being in direct contact with the growth medium in each well, necessitating its placement within the fluidic card. This requirement limits the sensor’s size to fit within the fluidic card’s dimensions of 7 cm by 10 cm. Additionally, to facilitate the flow of the liquid medium from the inlet to the outlet of the fluidic card, passing through each well, the sensor is designed with openings that match the diameter of the wells.

**Figure 4:**
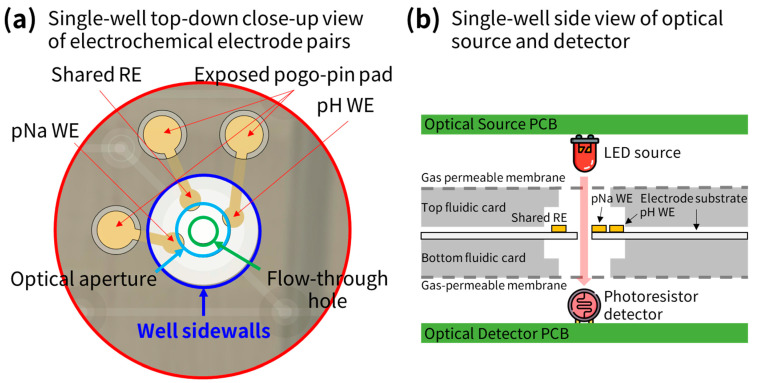
Close-up conceptual diagrams of the electrochemical and optical sensor setup. (**a**) Electrochemical electrode set. (**b**) Side-view of optical source and detector pair.

Furthermore, the multi-array sensor needs to connect with the source PCB, positioned right above the fluidic card. To effectively monitor the pH, pNa, and absorbance in each well, the sensor includes a pH-sensitive working electrode (WE), a pNa WE, and a shared reference electrode (RE). For optical measurements, an optical source (red LED, model no. KT-0603R, Hubei KENTO Electronic Co., Ltd., Yichang, China, typical emission wavelength at 620 nm [44]) is located on the source PCB above each well, and an optical detector (CdS photoresistor, model no. GL5537, Senba Sensing Tech, Nanyang, China, with peak 100% relative sensitivity at 540 nm and 80% relative sensitivity at 620 nm [45]) is located on the detector PCB beneath each well. Figure 4a,b provide a conceptual illustration of a single well, displaying the arrangement of the electrochemical electrodes (top-down view) and the optical absorbance sensor (side view).

Optical light sources (red LEDs) and detectors (CdS photoresistors) with peak emissions and high sensitivity around 600 nm were used due to the popular and well-known use of the standard OD_600_ measurements within the biology community. This method is a well-established, popular technique in the biological community for estimating cell concentration and growth, particularly in yeast cultures [46].

#### 2.2.2. Fabrication and Assembly

First, the tools used for electrode fabrication were as follows: the multi-array electrode was designed in AutoCAD (Autodesk, San Francisco, CA, USA) and fabricated in-house using laser-cut (Universal Laser Systems, Scottsdale, AZ, USA) thin polycarbonate film masks. Thermal and electronic beam evaporation (SHE-350-CR10, Samhan Membrane Vacuum, Gunpo-si, Republic of Korea) were used to deposit a thin Cr and Au film. The substrate of the multi-array electrode is a 250 μm-thick polycarbonate thin film with a 20 nm Cr adhesion layer and finally a 100 nm Au layer.

Next, the electrode layers were functionalized using the following processes. On top of the Au electrode layer, for the pH WE, PANI is electropolymerized using cyclic voltammetry (−0.2~1 V vs. commercial Ag/AgCl RE, Pt wire as CE, 100 mV step voltage, 40 cycles) in 0.1 M aniline and 1 M HCl solution. For the shared RE, Ag/AgCl ink is layered on top of the RE using a small toothpick. For the pNa WE, 2 μL of sodium ion selective membrane (made from ingredients given in Table 2) was dropcast on top of the pNa WE. Finally, the electrode was dried in a 1% relative humidity environment within a desiccator for 24 h before use.

After functionalizing all three electrodes, a laser-cut piece of 3M 300LSE pressure-sensitive adhesive (PSA) is employed for two purposes: as a passivation layer for the electrodes and as an adhesive layer between the electrode layer and the top and bottom fluidic layers. This PSA is precisely aligned with the electrode layer using four dowel pins located at each corner, and then it is firmly pressed down using a jig. Subsequently, this combined structure of the electrode layer and PSA is aligned with the top part of the fluidic card using the same four dowel pins and is manually pressed together. The same procedure is repeated for the bottom part of the fluidic card. This process marks the completion of integrating the multi-array electrode with the fluidic card.

#### 2.2.3. Performance Evaluation

To assess the functionality of the multi-array electrode sensor, two distinct experiments were conducted: one for the stability of the Ag/AgCl reference electrode and another for the pH and pNa sensor performance.

Firstly, for the stability and applicability of the Ag/AgCl reference electrode, its OCP output was compared to a commercial Ag/AgCl RE with a 3.5 M KCl filling solution in addition to acetate buffer solutions with different pH values (pH 6.0~4.0). Due to the challenges of connecting a commercial potentiostat to the actual multi-array electrode a stand-alone single-well electrode with easy access to all pH working and reference electrodes was used. Identical to the actual multi-electrode design, this stand-alone single-well electrode was composed of a PANI-based pH-sensitive WE, a shared Ag/AgCl RE, and a sodium-ionophore-based pNa-sensitive WE. The fabrication process was identical to the multi-electrode design described in Section 2.2.2. The Ag/AgCl RE’s OCP output (vs. commercial Ag/AgCl RE with 3.5 M KCl filling solution) in 3.5 M KCl was tested for 1000 s (≈16 min) to test for potential drift across time. Subsequently, the OCP of the Ag/AgCl electrode was tested across a pH range from 6.0 to 4.0, with each pH level maintained for 5 min, to determine any potential variations in response to different pH levels.

Secondly, the pH sensor experiment utilized sodium acetate buffer solutions with pH values from 5.6 to 3.6, while the pNa sensor experiment employed sodium chloride solutions with concentrations varying between 10^−5^ and 10^−1^ M. The complex design of the multi-array electrode (most electrodes could only be physically accessed via vertical PCB-mounted pogo-pins) posed a challenge in connecting a standard potentiostat to the numerous electrodes, particularly the shared reference electrode (RE). Consequently, for the evaluation of both pH and pNa sensors, a commercial Ag/AgCl RE containing a 3.5 M KCl solution was used alongside the respective pH and pNa electrodes, in combination with a commercial potentiostat. This potentiostat, set to open circuit potential (OCP) measurement mode, was utilized to record the OCP between the pH working electrode (WE) and Ag/AgCl RE, as well as between the pNa WE and Ag/AgCl RE.

### 2.3. On-Board Payload Computer

#### 2.3.1. Circuitry

The design and layout of the payload computer’s schematic and PCB layout were created using Kicad 6.0. The boards’ production was carried out by JLCPCB, which also soldered some of the components, while others were soldered in-house.

The entire schematics and overall explanation of each sheet for the OBPC are available within the SI (in Section S1.1., pages 1~7, Figures S1~S8). A visual and electrical board fabrication quality inspection was carried out and its results are included within Section S1.2 (Figure S9) and Section S1.3. in the SI (pages 7~8).

For all boards, the base material was a common FR4 with a glass transition temperature range of 135~140 °C. All had edge-cut dimensions of 110 mm × 70 mm (with a board outline tolerance of ±0.2 mm), with a board thickness of 1.6 mm, green PCB with white silk screen, tented via coverings, and an electroless nickel immersion gold (ENIG) surface finish.

**Figure 5:**
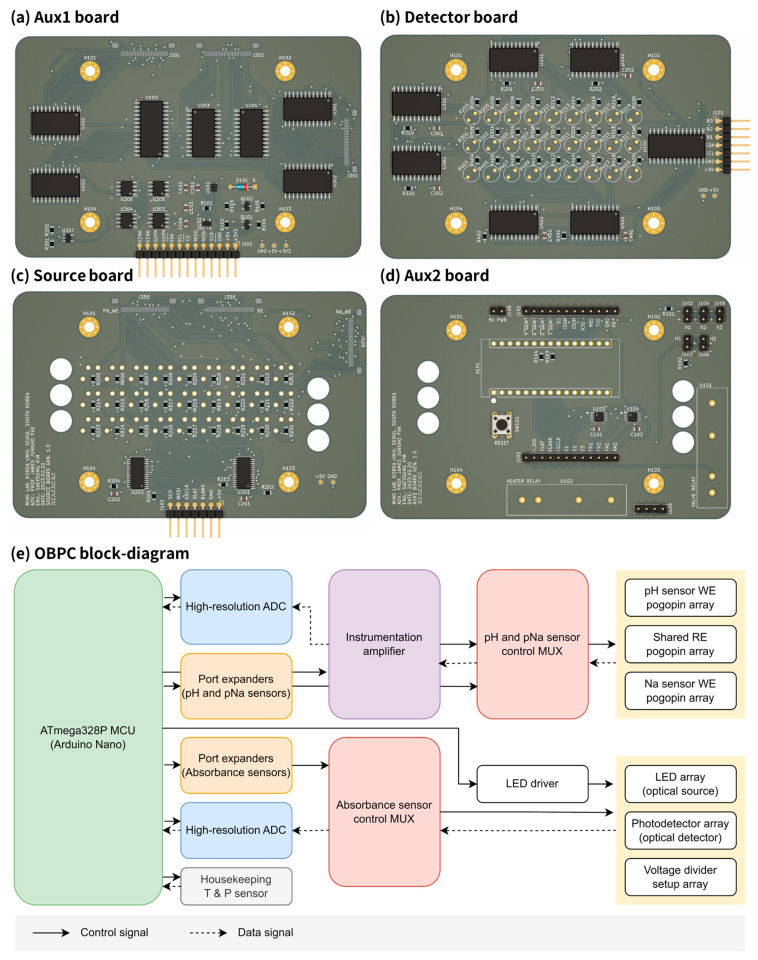
OBPC top-down view of (**a**) aux 1, (**b**) detector, (**c**) source, and (**d**) aux 2 board. (**e**) OBPC block diagram.

The on-board payload computer (OBPC) serves as the central processing unit for the entire payload system. Its primary functions include gathering data from the sensor readout circuits, processing these data, storing them, and periodically transmitting them to the on-board computer (OBC) located within the bus, external to the payload. Structurally, the OBPC comprises four printed circuit boards (PCBs) arranged in a stacked formation, with the fluidic card positioned centrally, flanked by two PCBs above and below. At the top of this stack is the Auxiliary board #2, as shown in Figure 5d, followed by the Source board just beneath it (Figure 5c). The fluidic card is then placed, followed by the Detector board (Figure 5b) directly underneath it. The stack concludes with the Auxiliary board #1 at the bottom, depicted in Figure 5a.

Here is an overview of the components on each board: the Auxiliary board #2 (Layer #4) contains the ATmega328P MCU, 2 high resolution ADS1115 ADCs, and various other external connectors (Figure 5d). The Source board (Layer #3) contains the light source LED array and the 81-pogopin array, 3 for each well (a total of 27 wells) (Figure 5c). The Detector board (Layer #2) contains the photodetector and voltage divider setup array for each well, 2 MUXs for each bank (a total of 3 banks, each bank composed of 9 wells) and a port expander (Figure 5b). Finally, the Auxiliary board #1 (Layer #1) contains a BMP280 temperature and pressure sensor, another port expander, 6 MUXs, and 4 instrumentation amps for the pH and pNa sensor readout circuit (Figure 5a).

The block diagram of the on-board payload computer (OBPC) is illustrated in Figure 5e. The sensors within the OBPC are primarily divided into two types: electrochemical sensors, which encompass the pH and pNa sensors, and optical sensors, represented by the absorbance sensors. Each type of sensor is distinguished by its unique interface components: the electrochemical sensors use electrodes and pogo-pin arrays, while the optical sensors employ an optical source and detector. Both sensor categories make use of multiplexers (MUXs), port expanders, and high-resolution analog-to-digital converters (ADCs). Additionally, the electrochemical sensors incorporate instrumentation amplifiers, and the optical sensors are fitted with LED driver chips.

**Figure 6:**
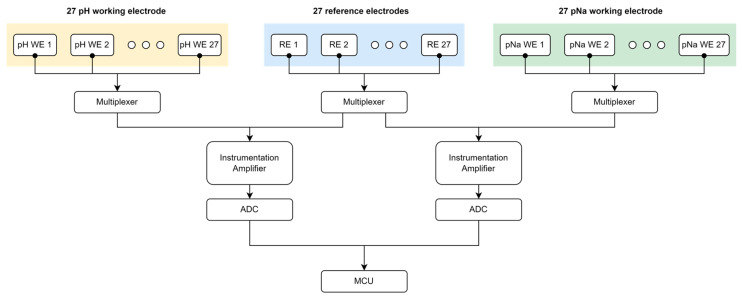
Multi-modal multi-array electrochemical pH and pNa sensor electrode OCP measurement circuitry block diagram.

Figure 6 delineates the block diagram of the multi-array electrochemical pH and pNa sensor electrode open-circuit potential (OCP) measurement circuitry which orchestrates data acquisition from a suite of electrochemical sensor electrodes. The electrochemical subset comprises 27 pH and pNa working electrodes (WE), each juxtaposed with a corresponding reference electrode (RE) across a series of multiplexers. These multiplexers facilitate the sequential selection of signals from the WE and RE, routing them through instrumentation amplifiers to bolster signal integrity before conversion by high-resolution analog-to-digital converters (ADCs). The ADCs digitize the amplified signals for subsequent processing by the microcontroller unit (MCU), which governs the multiplexer control logic, data interpretation, and communication protocols.

**Figure 7:**
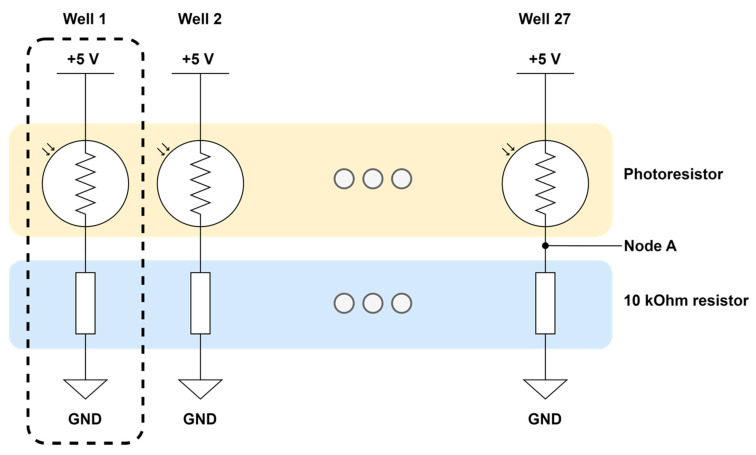
Optical detector voltage divider array diagram.

Figure 7 depicts the optical detector voltage divider array for the optical absorbance sensor. Each voltage divider is powered by +5 V and has a photoresistor and a 10k resistor in a voltage divider configuration. There are a total of 27 voltage dividers, one for each well. Voltage measurements are taken from in-between the photoresistor and 10k resistor, noted as Node A in Figure 7. An increase in light intensity on the CdS photoresistor surface would result in a decrease in photoresistor resistance, which in turn would increase the measured voltage at Node A.

**Figure 8:**
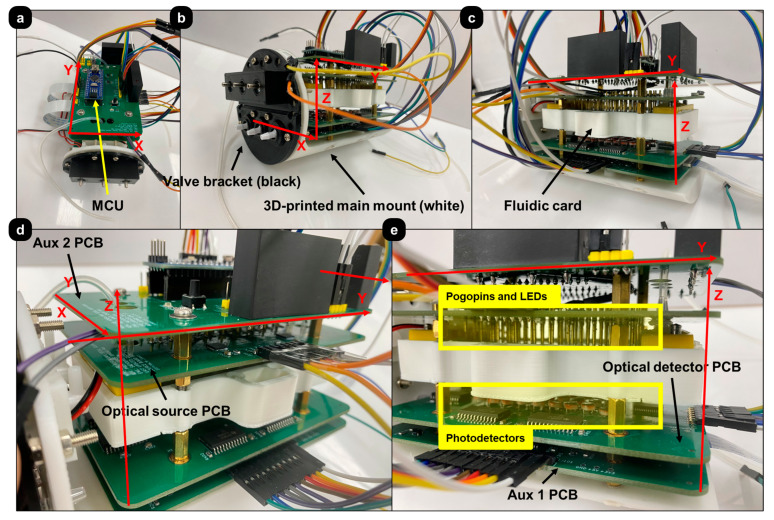
Assembled OBPC development model. (**a**) MCU ATmega328P on top of the OBPC top board. (**b**) Black valve bracket and white main mount. (**c**) Fluidic card and its 3D printed mount sandwiched between the 4 OBPC PCBs. (**d**) Close-up view of the PCB stack. (**e**) Side-view of the PCB stack showing the pogo-pin array protruding from the source board and extending down into the fluidic card.

Figure 8 shows multiple photographs from different angles of an assembled OBPC development model. The process is explained step-by-step in Section S3 of the SI (Figure S12).

#### 2.3.2. Firmware

The functional operation of the on-board payload computer’s (OBPC) PCB stack is facilitated by its bespoke firmware, referred to as the OBPC firmware (FW). This firmware’s primary objective is threefold: firstly, to ascertain the voltage readings from the circuits dedicated to pH, pNa, and absorbance; secondly, to record these data within the on-board static random-access memory (SRAM) as variables; and thirdly, to transmit these data via the serial port to a personal computer for subsequent preservation and analysis.

Crafted in C++ utilizing the Arduino IDE version 2.2.1 on a Windows 10 platform, the firmware employs avr-gcc as its compiler. The simplicity and directness of the firmware negated the necessity for a debugger; instead, outputting to the serial monitor sufficed for any requisite troubleshooting. Version control was managed with Git.

**Figure 9:**
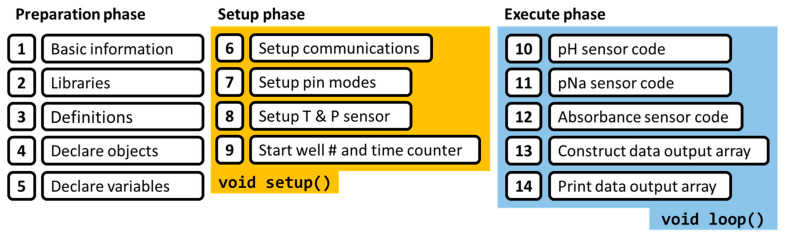
Firmware structure: preparation, setup, and execution.

As delineated in Figure 9, the firmware’s architecture is partitioned into three sequential phases: preparation, setup, and execution. The preparation phase encompasses initial code annotations, library incorporation, the definition of symbolic constants and macros, object instantiation, and variable declaration. The setup phase involves the configuration of communication protocols including serial, SPI, and I2C; the initialization of pin modes for the microcontroller unit (MCU) pins and port expanders, designated as outputs; and the configuration of the BMP280 sensor for ambient temperature and pressure monitoring, coupled with the initiation of counters for well enumeration and temporal tracking. The execution phase is dedicated to the operational tasks of measuring and compiling the pH, pNa, and absorbance data into a structured array of 85 elements, which is then dispatched to the serial monitor for display and logging, primed for further analytical processing. Further detailed code snippets and the firmware output are available in the SI in Section S2 (Figures S10 and S11, pages 8~10).

#### 2.3.3. OBPC Functional Testing

The primary objective of the payload is to determine the pH, pNa, and absorbance within each of the 27 wells on the fluidic card. Consequently, the integrated system comprising the on-board payload computer (OBPC) and the fluidic card is subjected to evaluation based on three parameters: the outputs of the pH sensor, pNa sensor, and absorbance sensor.

Prior to conducting measurements using various liquid samples for different pH, pNa, and absorbance levels, initial tests were carried out on the OBPC sensor module to validate its input and output functions. These preliminary tests were of two types. The first test involved assessing the electrochemical pH and pNa sensor modules by substituting the electrodes with a power supply unit (PSU) to feed input voltages ranging from 0 to 500 mV and 0 to −500 mV and observing the corresponding outputs on the OBPC to ensure its proper operation. The second test for the optical absorbance sensor involved covering the photodetector arrays with optically opaque black matte sandpaper to verify that diminished light incidence resulted in an increased resistance measurement, with all tests being performed in conditions mimicking a dark room.

Regarding the liquid test samples, pH calibration buffer solutions with known pH values of 10.00, 7.00, and 4.00 (Orion™ pH buffer bottles, Thermo Fisher Scientific, Waltham, MA, USA) were utilized to establish a calibration curve correlating pH to open circuit potential. For the pNa sensor, solutions of sodium chloride with concentrations from 10^−5^ M to 10^−1^ M were prepared. Absorbance sensor evaluations employed suspensions of silica (SiO_2_) microspheres, chosen for their uniform absorbance properties and ubiquity in testing absorbance sensors, with diameters ranging from 9 to 13 μm and concentrations from 0 to 10 g/L. These suspensions, serially diluted in deionized water to create a range of turbidity corresponding to different concentrations, were introduced into the fluidic card using an infusion pump at a rate of 1 mL/min to a total volume of 3 mL. The turbidity levels, indicative of the silica microspheres’ concentration, directly influenced the sample’s absorbance, with denser suspensions exhibiting higher absorbance values.

The raw voltage outputs from the sensors were recorded and converted to pH, pNa, and absorbance values. For pH, a linear equation was derived from voltage readings at pH 10, 7, and 4, with the formula y=ax+b, where ‘a’ is the slope adjusted for the circuit’s gain of 5, yielding the actual sensor sensitivity as 1/5 of ‘a’. This method was also used to calculate the sensitivity for the pNa sensor.

A diagram of the photoresistor and voltage divider array of the optical absorbance sensor is available in Figure 7. For the absorbance values, first, the voltage value ($V_{A}$) at Node A (Figure 7) is measured by the OBPC optical absorbance sensor. Since it is a voltage divider configuration, the following equation holds true.

(1) $$R=10^{4}\times \left(5.0-V_{A}\right)/\left(V_{A}\right)$$

where $V_{A}$ is the measured voltage value at Node A in volts (V) and R is the photoresistor resistance in ohms (Ω). This equation is then used to convert from measured voltage to the resistance of the photoresistor.

The transfer function between the measured photoresistor resistance and light intensity is given as follows, according to the GL5537-2 photoresistor datasheet:

(2) $$I=3.24358\times 10^{7}/R^{1.490}$$

where R is the photoresistor resistance in ohms (Ω) and I is the corresponding light intensity in lux.

Once the light intensity is calculated, the Beer–Lambert law can be used to calculate absorbance. The relationship between measured absorbance and analyte concentration is described by the Beer–Lambert law (which assumes no dispersion, no reflection, and no scattering) as:

(3) $$T\left(\%\right)=\left(\frac{I}{I_{0}}\right)\times 100$$

(4) $$A=\varepsilon cl=\log_{10}\frac{I_{0}}{I}=\log_{10}\frac{100}{T}$$

where T is the optical transmittance (%), $I_{0}$ is the light intensity of incident light (or the intensity of the blank/reference solution), I is the light intensity after passing through the sample (or with the particle of interest within the blank/reference solution), A is the light absorbance of the particles (dimensionless), ε is the molar attenuation coefficient (M^−1^cm^−1^), c is the molar concentration (M), and l is the optical path length (cm).

## 3. Results and Discussion

### 3.1. Fluidic Card Flow and Leakage Test Results

The fluidic card underwent rigorous evaluation against a set of stringent leakage criteria, encompassing intra-bank, inter-bank, and overall leakage parameters. The intra-bank assessment scrutinized potential liquid transference between wells within the same bank or into ancillary features such as pogo-pin holes. The inter-bank analysis examined the containment integrity between distinct banks, ensuring no cross-contamination occurred. Lastly, the overall leakage inspection ensured that the fluid contained within the wells and channels remained securely within the card’s boundaries, with no escape from the periphery. The card was able to sustain an infusion rate of up to 1 mL/min without any sort of leakage.

**Figure 10:**
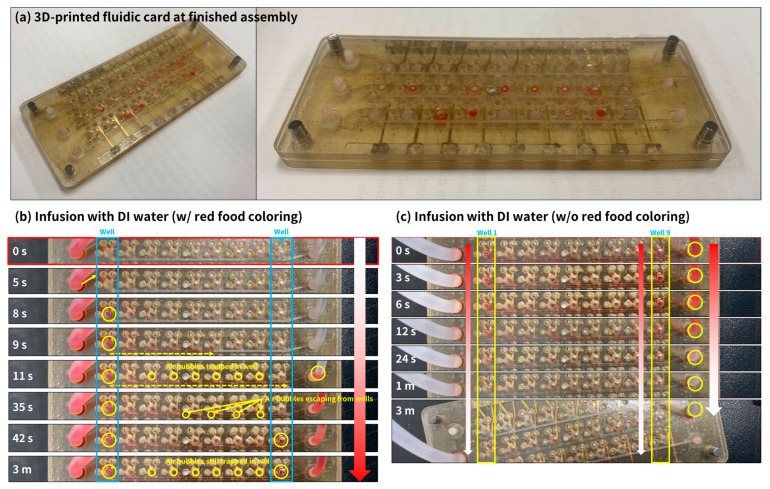
Photograph of assembled fluidic card and flow and leakage test results. (**a**) Photograph of assembled fluidic card. (**b**) Timelapse of infusion with DI water with red coloring. (**c**) Timelapse of infusion with DI water without red coloring.

Nonetheless, the filling of the wells presented challenges, with only wells #1 and #9 of bank 2 (flow and leakage was tested on bank 2, the most susceptible to leakage among the three banks) receiving liquid while the others remained unfilled, as depicted in Figure 10 (Figure 10b, yellow circle on wells #1 and #9, starting at 8 s for #1 and 42 s for #9). This pattern indicates that the individual flow capacity of each well was sufficient to handle the infusion rate of 1 mL/min. Consequently, rather than sequential filling from wells #1 to #9 or vice versa, the liquid would fill a single well and the primary outlet channel. This process caused air to be entrapped in the intermediate wells (#2 to #8), a phenomenon observable in Figure 10b at the 35 s mark, where air bubbles were seen intermittently rising from the wells into the outlet trunk channel. Subsequent to this, fluid flow was restricted to only wells #1 and #9. This behavior also suggests that the fluidic resistance was minimal for these two wells, which both managed to accommodate the full infusion rate without any leakage. It is important to note that during these observations, the liquid consistently flowed through the inlet, into the well, and out through the outlet.

For future work, several recommendations are proposed to address the well fill problem observed in the fluidic card. Redesigning the fluidic pathways could ensure a more equitable liquid distribution to each well. Introducing air vents might facilitate the release of entrapped air, aiding in efficient filling. A sequential filling strategy and varying infusion rates should be explored to optimize liquid delivery. The application of hydrophilic coatings could reduce surface tension, enhancing well filling. Additionally, pressure modulation during infusion may overcome fluidic resistance. Utilizing computational fluid dynamics (CFD) simulations could provide valuable insights for design improvements. Finally, integrating real-time feedback mechanisms, such as sensors to monitor well filling, could dynamically adjust the flow. These approaches offer potential avenues for the future refinement of the fluidic card’s design and functionality.

### 3.2. Multi-Modal Multi-Array Electrochemical Electrode Performance Evaluation

#### 3.2.1. Stand-Alone Single-Well Ag/AgCl RE Evaluation

The stability and applicability of the Ag/AgCl reference electrode is shown in full detail in Figure S13 of the supporting material. First, its stability was evaluated and its OCP output (vs. commercial Ag/AgCl RE) across 1000 s is shown in Figure S13a. The custom Ag/AgCl pseudo-reference electrode (RE) demonstrated consistent performance, maintaining a potential of −13.4 ± 0.9 mV (mean ± standard deviation) when compared to a standard commercial Ag/AgCl reference electrode over a duration of at least 15 min. This indicates the potential of the fabricated Ag/AgCl pseudo-RE to serve as a viable alternative to commercial Ag/AgCl REs, with the ability to sustain a steady potential over a time period without notable deviation.

Additionally, its stability in varying pH levels ranging from pH 6.0 to 4.0 is shown in Figure S13b. The output OCP displayed no significant increase or decrease in response to pH variations, showing a stable output potential of 2379.4 mV on average. This shows that the Ag/AgCl RE maintained a stable potential even at different pH levels.

The experimental results for Ag/AgCl RE potential stability across time and pH levels showed that the Ag/AgCl RE was suitable as an electrochemical reference electrode.

#### 3.2.2. Multi-Array Electrochemical Electrode Evaluation

**Figure 11:**
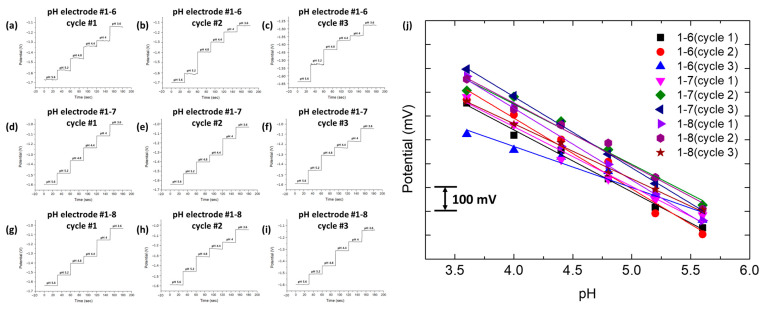
Multi-array pH electrode OCP response. (**a**–**c**) pH electrode #1-6. (**d**–**f**) pH electrode #1-7. (**g**–**i**) pH electrode #1-8. (**j**) Average and linear fit plot of each pH electrode and cycle.

Figure 11 illustrates the open circuit potential (OCP) measurements for three pH electrodes (#1–6, #1–7, and #1–8) within sodium acetate buffer solutions spanning pH values from 5.6 to 3.6. Each electrode, representing the sixth, seventh, and eighth electrodes in bank one, respectively, underwent OCP assessments for 30 s at each pH level.

The average OCP for each pH was determined over three trials and is graphically represented in Figure 11j, along with a linear fit line. The linearity of the response was confirmed by an average R-squared value of 0.991, indicating consistent performance across the tested pH range. These findings validate the design, fabrication, and functionality of the multi-array pH electrodes.

**Figure 12:**
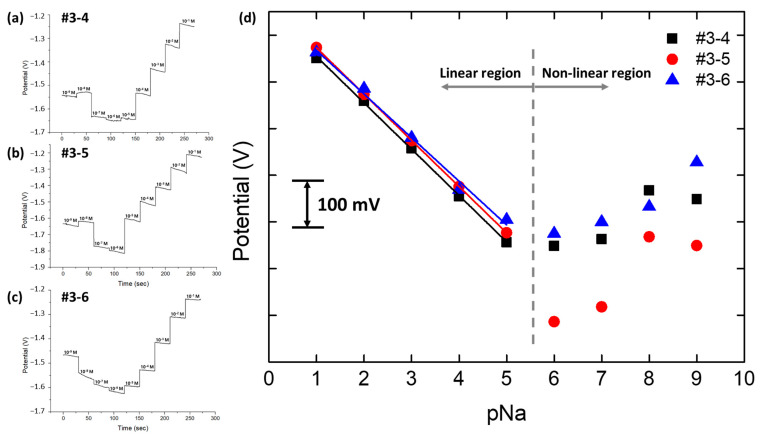
Multi-array pNa electrode OCP response. (**a**) pNa electrode #3-4. (**b**) pNa electrode #3-5. (**c**) pNa electrode #3-6. (**d**) Average and linear fit plot of each pNa electrode.

Figure 12 presents the open circuit potential (OCP) measurements for three pNa electrodes (#3-4, #3-5, and #3-6), immersed in sodium chloride (NaCl) solutions with concentrations from 10^−9^ M to 10^−1^ M. OCP readings were taken for 30 s at each concentration level. It is important to note that the concentration of sodium ions can be expressed as pNa values, where pNa is the negative logarithm to the base ten of the sodium ion concentration, hence ranging from pNa 9 to pNa 1 for the given molarities. OCP readings for concentrations corresponding to pNa greater than 6 (concentrations below 10^−5^ M) did not follow the linear relationship. However, for concentrations represented by pNa less than 5, the electrodes exhibited a consistent linear response with high R-squared values exceeding 0.998. These data indicate that the multi-array pNa electrodes are operating effectively as per their design, and were suitable for integration with the OBPC for final testing.

### 3.3. OBPC Functional Test Results

**Figure 13:**
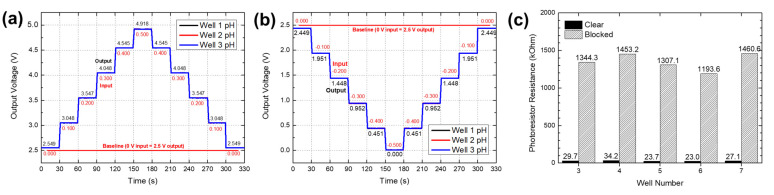
OBPC electrochemical sensor readout circuit output voltage according to (**a**) positive and (**b**) negative input voltage. (**c**) Photoresistor resistance value change depending on whether or not the optical pathway to the photoresistor is blocked.

First, results from the initial testing are presented in Figure 13. The electrochemical pH and pNa sensor modules were evaluated, as depicted in Figure 13a,b, by interfacing the inputs of their open circuit potential readout circuits with a power supply unit (PSU). It was observed that an increment in the PSU’s output from 0 to 500 mV (in 100 mV steps) corresponded to an increase in the readout circuit’s output from 2.549 V to 4.918 V (in 500 mV increments). Conversely, a decrease in the PSU’s output from 0 to −500 mV (in −100 mV steps) resulted in a reduction in the readout circuit’s output from 2.449 V to 0 V (in 500 mV decrements). These outcomes were in line with the transfer function of the electrochemical pH and pNa readout circuit, characterized by a voltage output of 5 times the voltage input plus a 2.5 V DC offset ($V_{out}=5V_{in}+2.5$). The findings confirmed that the electrochemical pH and pNa readout circuit within the OBPC operated correctly and as intended.

Second, the examination results for the optical absorbance sensor, involving the use of optically opaque black matte sandpaper to obstruct the photodetector arrays, are documented in Figure 13c. This test was conducted to verify that diminished illumination on the photodetector arrays would lead to an elevated resistance measurement. Initially, when the photoresistor array was unobstructed, allowing unimpeded light from the source PCB to reach the photoresistors through the fluidic card, the photoresistors exhibited an average resistance of approximately 27.5 ohms. Conversely, when the array was covered with black matte sandpaper, the average resistance value rose to 1351.4 ohms. This experiment utilized absorbance sensors from five wells, specifically bank 1 wells 3 through to 8, due to the limited physical accessibility of the remaining wells when the fluidic card was assembled with the OBPC.

**Figure 14:**
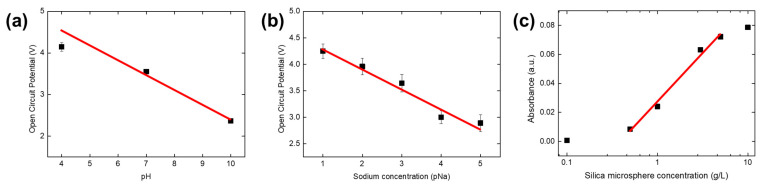
Test results using actual liquid samples. (**a**) pH sensor output OCP in various pH buffer solutions. (**b**) pNa sensor output OCP in various sodium chloride concentration solutions. (**c**) Absorbance sensor output in various silica microsphere dilutions.

Figure 14 presents the test outcomes from employing actual liquid samples in sensor experiments: pH buffer solutions for the pH sensor (Figure 14a), sodium chloride solutions for the pNa sensor (Figure 14b), and silica microsphere dilutions for the absorbance sensor (Figure 14c). The results are displayed for bank 3 well 19’s pH and pNa sensors in Figure 14a,b, as this was the only well that was successfully filled with the test liquids. The output from the pH and pNa sensors exhibited transfer functions of $V_{OCP}=-0.355\left(pH\right)+5.952$ ($R^{2}=0.986$) and $V_{OCP}=-0.376\left(pNa\right)+4.651$ ($R^{2}=0.979$), respectively. These functions translate to pH and pNa sensitivities of 355 mV/pH and 376 mV/pNa, with an actual sensitivity of 71.0 mV/pH and 75.2 mV/pNa after accounting for the gain of 5. Such supra-Nernstian responses align with expectations for PANI-based pH sensors and sodium ionophore X Selectophore™-based sodium sensors [40,41,42,43]. Regarding the absorbance sensor, as shown in Figure 14c, the absorbance varied with SiO_2_ concentrations ranging from 0 to 10 g/L. A linear regression analysis from 0.1 to 5 g/L yielded a linear fit function $A=0.067\log_{10}c+0.027$ ($R^{2}=0.994$). The linearity of the sensor outputs, all showing adequate sensitivity levels and exceeding 0.99 R-squared values, confirmed the functionality of the development model post-assembly, integration, and testing (AIT) procedures.

### 3.4. Discussion and Potential Future Work

The intricacies of fluid dynamics within the fluidic card, particularly the well fill problem, emerged as a critical area for future improvement. The observed preferential filling of specific wells underlines the need for a more refined fluidic design. Future work could involve redesigning the internal channel architecture and introducing air vents or hydrophilic coatings to mitigate air entrapment and ensure a uniform liquid distribution across all wells. Additionally, exploring the use of pressure modulation or varying infusion rates could further optimize fluid delivery.

Subsequent research should also delve into the long-term stability and reliability of these pH, pNa, and absorbance sensors in space-like conditions, including exposure to microgravity, radiation, and thermal fluctuations. This could involve extended testing on the ground or in low Earth orbit, potentially on the ISS or other CubeSat platforms, as a technology demonstration mission. The integration of more advanced data processing algorithms and real-time monitoring systems would enhance the operational efficiency and autonomy of the sensor suite.

Furthermore, addressing the challenges posed by the CubeSat’s limited power and thermal management capabilities will be crucial. Optimizing the power consumption of the sensor module and exploring passive or active thermal control strategies would be key areas of focus.

## 4. Conclusions

In conclusion, the assessment of the CubeSat’s development model during the assembly, integration, and testing (AIT) phase has conclusively demonstrated the successful assembly and functional cohesiveness of the subsystems, which include the fluidic card, multi-modal multi-array electrode, and OBPC. The presented multi-model multi-array electrochemical and optical sensor suite showed a dependable transfer function with electrochemical pH and pNa sensors delivering sufficient response and confirming the circuitry’s precision as per design expectations, notably with a linear transfer function. Optical tests also affirmed the absorbance sensor’s functionality, with dramatic resistance shifts upon light path obstruction. The pH and pNa sensors showed supra-Nernstian responses (71 mV/pH and 75.2 mV/pNa) and high linearity (>0.99) as expected, while the absorbance sensor displayed a highly accurate turbidity measurement capability across varying concentrations of silica microspheres (0.1~5 g/L), as evidenced by a sensitivity of 0.067 AU and an R-square value of 0.994. This research has validated the functional readiness of the CubeSat’s development model and suggests a positive operational forecast for its deployment in space after further ground level tests such as vibration, thermal, and radiation testing. The data from this research phase underpin the multi-modal multi-array sensor module’s potential for applications in space-based biology experiments and provide a solid foundation for future explorations and payload diagnostics in space missions.

## Figures and Tables

**Table 1:** Historical CubeSats with their target organisms and measurement capabilities.

CubeSat Name	Organism	Size	Measured Parameters	Year	Ref.
GeneSat-1	*E. coli*	3U	Optical absorbance	2006	[13,14,15]
PharmaSat 1	*S. cerevisiae*	3U	Optical absorbance	2009	[16,17,18]
O/OREOS	*B. subtilis*	3U	Optical absorbance	2010	[19,20,21,22]
SporeSat-1	*Ceratopteris richardii*	3U	Calcium ions	2014	[31]
GraviSat	Cyanobacteria/Algal Cultures	3U	Optical absorbance	Not launched	[32]
SporeSat-2	*Ceratopteris richardii*	3U	Optical absorbance	Not launched	[33]
EcAMSat	*E. coli*	6U	Optical absorbance	2017	[23,24,25]
BioSentinel	*S. cerevisiae*	6U	Optical absorbance	2022	[26,27,28,29,30]

**Table 2:** Ingredients for sodium ion selective membrane cocktail.

Ingredient	%	Weight or Volume
Sodium ionophore X	1 wt%	18.18 mg
sodium tetrakis [3,5 bis(trifluoromethyl)phenyl] borate (Na-TFPB)	0.55 wt%	10 mg
polyvinylchloride (PVC)	33 wt%	600 mg
bis(2-ethylehexyl) sebacate (DOS)	65.45 wt%	1.3 mL
tetrahydrofuran	660 uL	12 mL

## Data Availability

Data contained within the article.

## Supplementary Materials

The following supporting information can be downloaded at: https://www.mdpi.com/article/10.3390/s24010265/s1.
