# Supplementary material for: Multi-Modal Multi-Array Electrochemical and Optical Sensor Suite for a Biological CubeSat Payload
Source: Sensors (Basel). 2024 Jan 2;24(1):265. doi: 10.3390/s24010265 (PMC10781281; doi:10.3390/s24010265)
Supplement: Supplementary file 1 [file sensors-24-00265-s001.zip › sensors-2744976-supplementary.pdf]

Supplementary material

1. Circuitry design and fabrication

1.1. Schematics

In this section each schematic for each board will be explained in detail. Figure S1 shows the top-level schematic for Auxiliary #1 board. It's sub-level schematics for the pH sensor and pNa sensor portion are shown in Figure S2. A port expander responsible for the control of MUXs interfacing with the electrochemical sensors is placed here (shown on the right side of Figure S1) along with the BMP280 temperature and pressure sensor for housekeeping. The port expander interfaces with the MCU via I^{2}C and controls the MUXs via 4 digital select lines for each MUX. The 2 squares on the bottom left are hierarchical Kicad sheets for the pH and pNa sensor schematic. Double-clicking them would open a new sub-level sheet that is nested within the original top-level sheet.

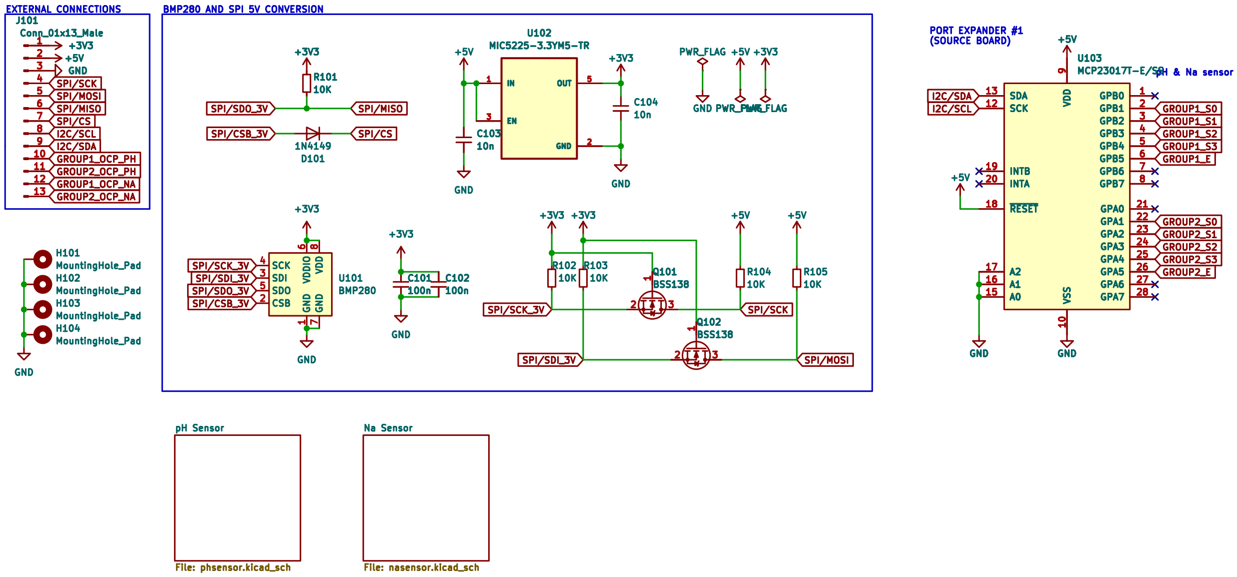

Figure S1. Top-level schematic for Auxiliary #1 board.

Figure S2 and S3 show the sub-level sheets for the pH and pNa sensor portion. There are 27 wells, and for each well, there is a pH WE (working electrode), pNa WE, and shared RE (reference electrode). In the sub-level sheet for the pH sensor portion (Figure S2), 27 pogo-pins for the pH WE and shared RE are shown as connectors on the right. These pins are connected to the 4 MUXs on the left. Then the COM pins on these MUXs are connected to the instrumentation amp (AD8223) for open circuit potential (OCP) measurements. The final OCP measurements are relayed to the ADC, not shown in Figure S2 and S3. The overall flow of data and connections are the same for the pNa sensor portion shown in Figure S3.

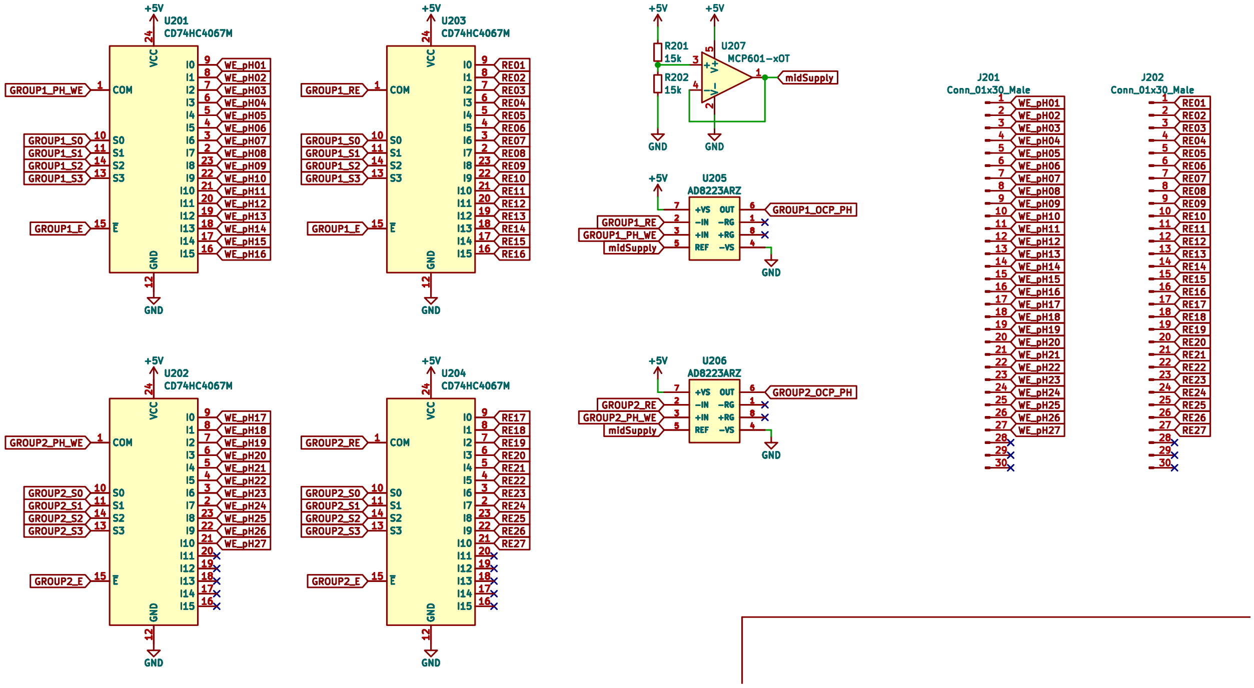

Figure S2. Sub-level schematic for Auxiliary #1 board pH sensor section.

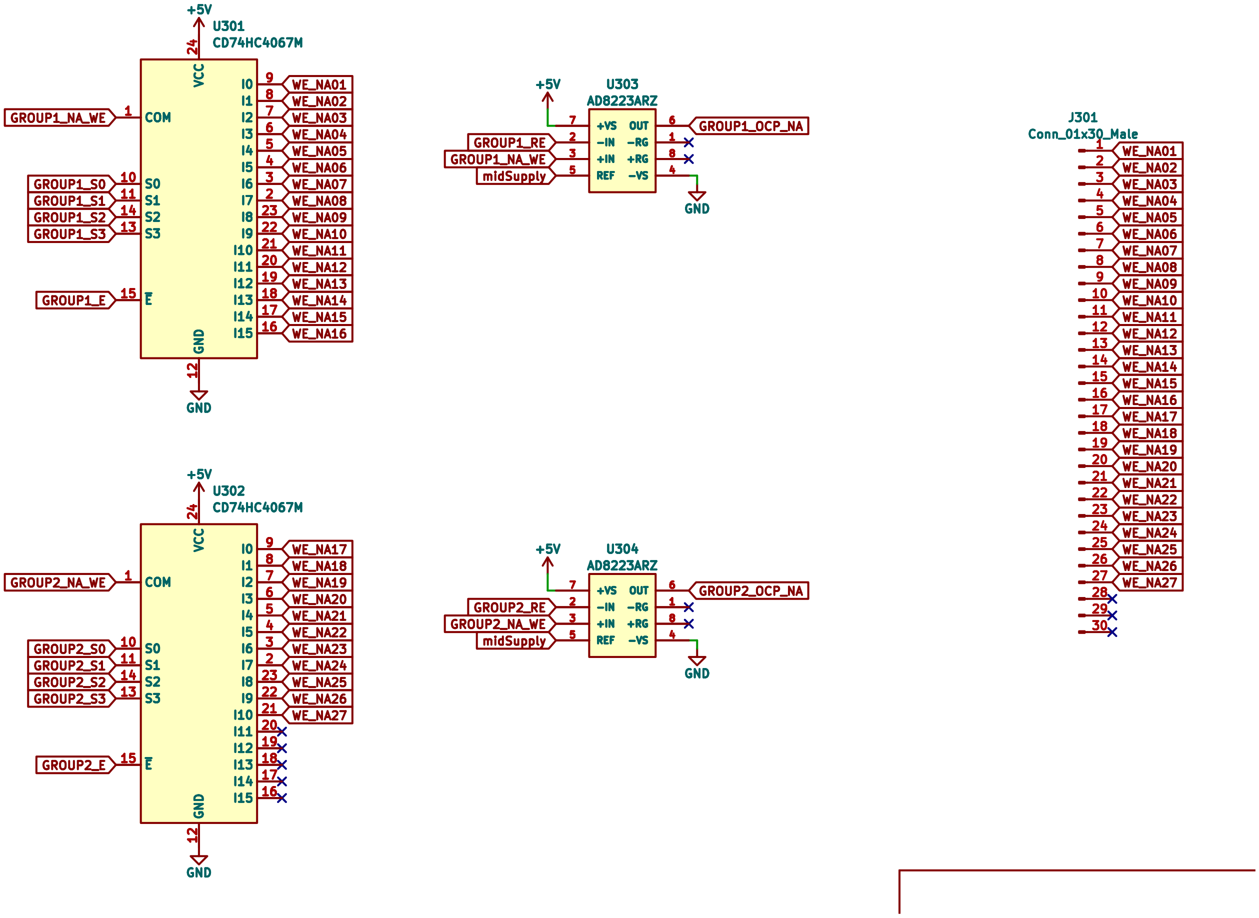

Figure S3. Sub-level schematic for Auxiliary #1 board Na sensor schematic.

Figure S4 shows the top-level schematic for the Detector board (Layer #2). A port expander for MUX control of Bank 1, 2, and 3 of the optical absorbance sensor is on the top right side of the sheet and interfaces with the MCU through I2C. On the left are 3 sub-level sheets for the MUX and photodetector voltage divider array of bank 1, 2 and 3, shown in Figure S5. As can be seen in Figure S5, on the left are 2 MUXs, and on the right are 9 pairs of voltage dividers, 1 pair of photodetector and resistor for each well.

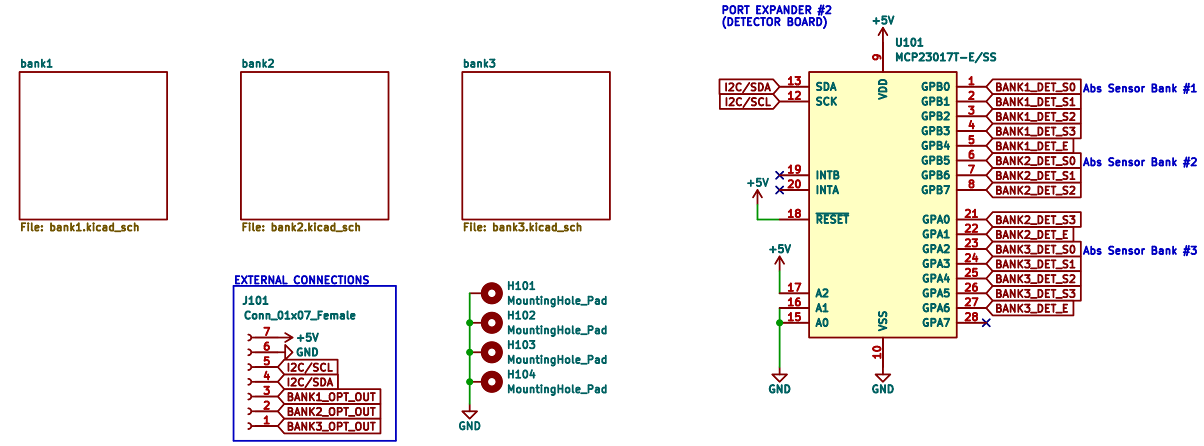

Figure S4. Top-level sheet for detector board schematics.

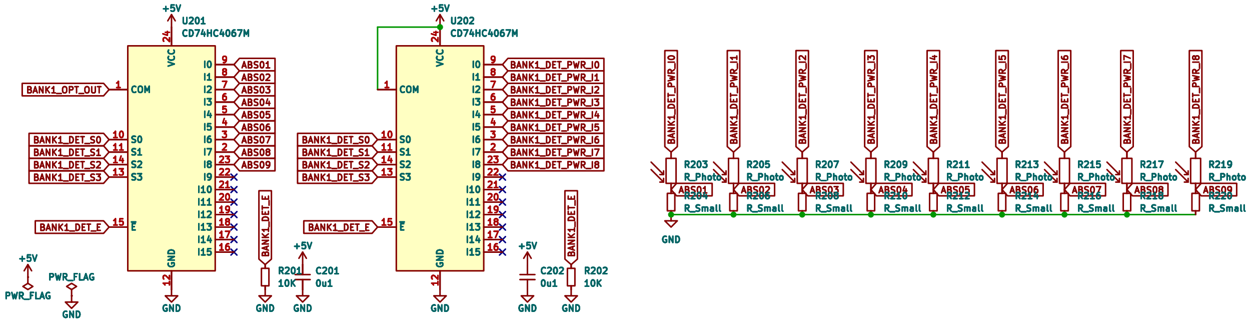

Figure S5. Sub-level sheet for detector board bank 1 (the same for Bank 2 and 3).

Figure S6 shows the top-level schematic for the Source board (Layer #3). Here, on the bottom is a connector for SPI interfacing with the main MCU, and on the top are 3 sub-level sheets for the optical, pH, and pNa sensor circuit. For the pH and pNa portion (not shown), there are connector symbols for 3× 27 pogo-pins each: 27 pH sensor WEs, 27 pNa sensor WEs, and 27 shared REs. For the optical absorbance sensor portion, shown in Figure S7, 27 LED arrays are connected to 2 LED driver chips. One chip responsible for controlling the first 16 LEDs (from well 1 ~ 16), another for the remaining 11 LEDs (well 17 ~ 27). The two LED driver chips are daisy chained and interface with the MCU through SPI.

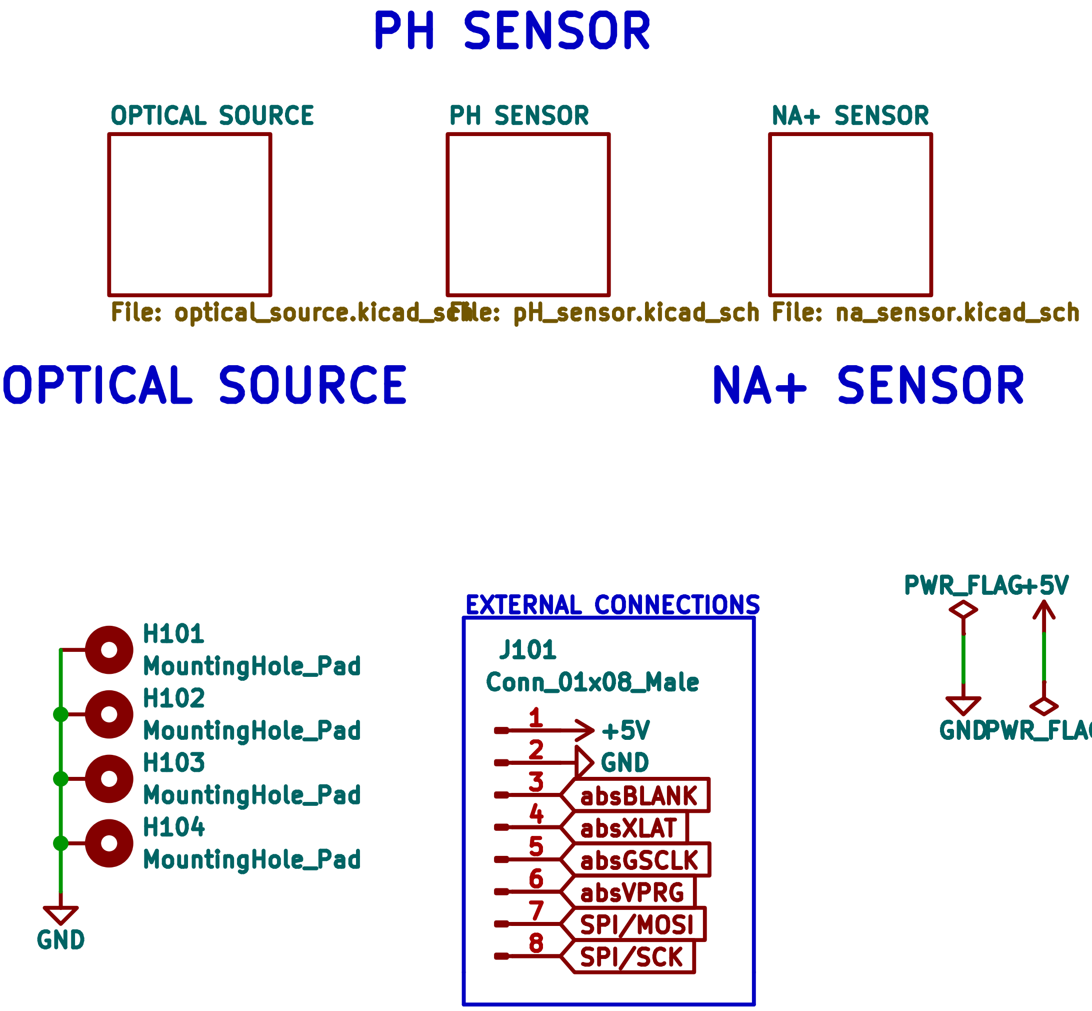

Figure S6. Top-level sheet for source board schematic.

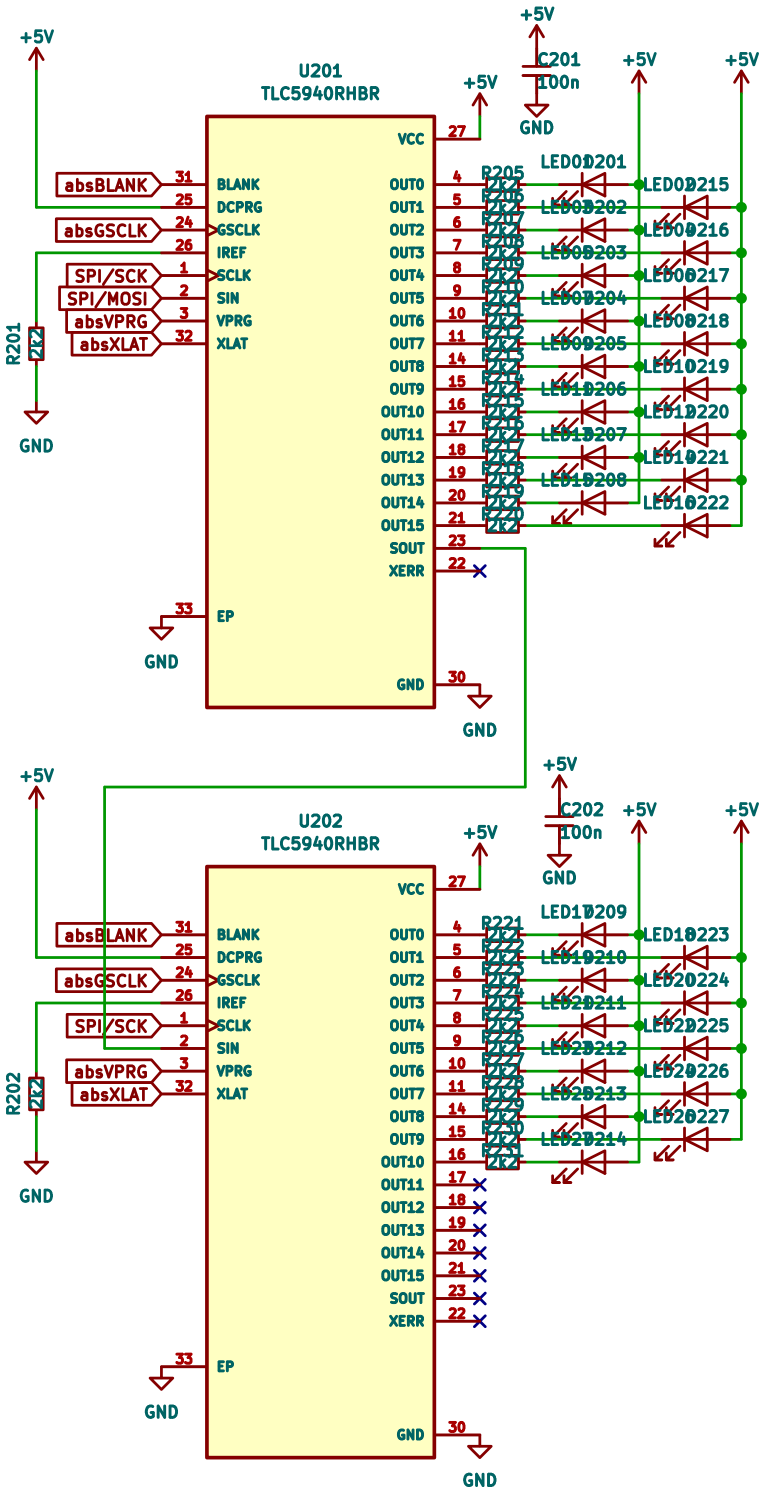

Figure S7. Source board LED driver schematic.

Lastly, the final Aux 2 board (Layer #4) schematic is given in Figure S8. On here, the major components are the Arduino Nano (ATmega328P) MCU, 2 high resolution ADCs, and all the connectors that connect this board with the rest of the boards. The 2 high resolution ADCs interface with the MCU by I2C and each ADC receives voltage data from the optical and electrochemical sensor readout circuits, respectively.

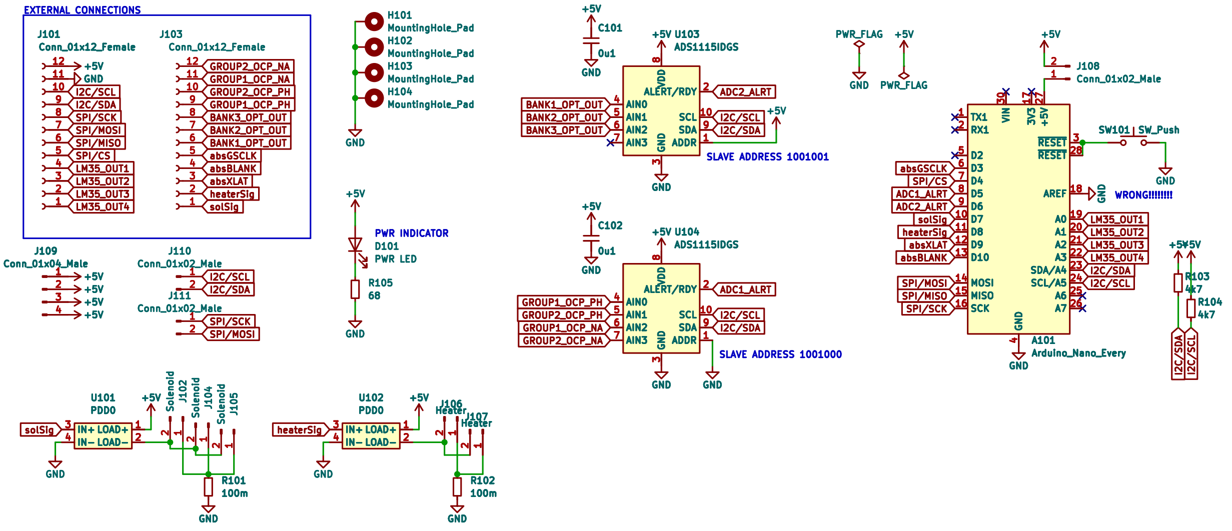

Figure S8. Aux 2 board schematic.

1.2. Visual inspection of the PCBs

In the visual inspection of PCBs, soldering was carefully checked for defects, components were verified for correct placement, and traces and pads were inspected for damage. The boards were examined for cleanliness, physical integrity, and any signs of etching errors. Drill alignment, solder mask uniformity, and the clarity of printed legends were also assessed. Plating on through-holes and edge connectors was evaluated for consistency. Multilayer boards were checked for layer alignment and via integrity. Photographs of finished PCBs are shown in Figure S9.

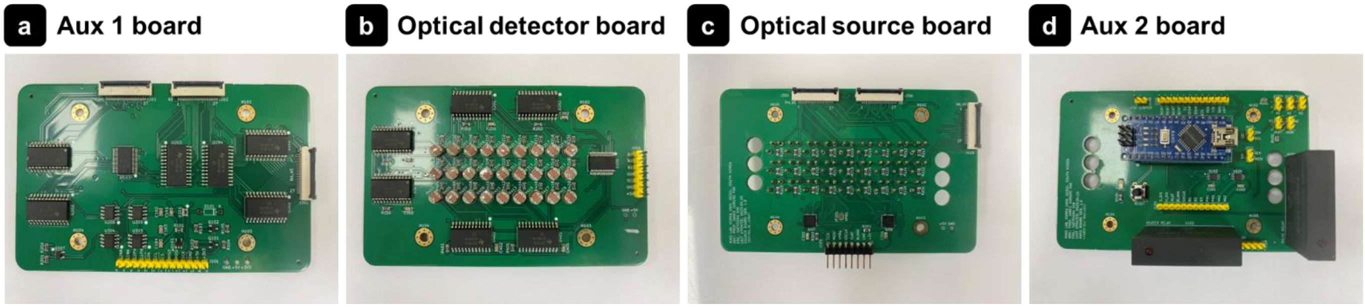

Figure S9. Photograph of assembled PCBs received from JLCPCB using their SMT assembly service and further soldering done in-house. Top-down view of (a) Aux 1 board, (b) optical detector board, (c) optical source board, and (d) Aux 2 board.

1.3. Continuity, power-up, and voltage level tests

During the continuity testing of PCBs, electrical paths were confirmed for conductivity against the schematic, with unintended connections being probed for potential short circuits. Open circuits were identified by detecting breaks in continuity, particularly in traces and unsoldered connections. Test points and vias were evaluated to verify their correct integration into the circuit, especially for ensuring interlayer connectivity in multi-layer PCBs.

The PCBs were powered with a +5 V power supply unit (PSU) and voltage levels at test points (+5 V, +3.3 V, and ground), connectors, vias, and plated-mount holes all returned correct voltage values.

2. Firmware

2.1. Code

Figure S10b and S10c detail the code segment accountable for capturing the output voltage from the pH sensor's readout circuit. This code segment, which is representative of the coding approach for the pH, pNa, and absorbance sensors, is featured prominently at the commencement of the void loop() function.

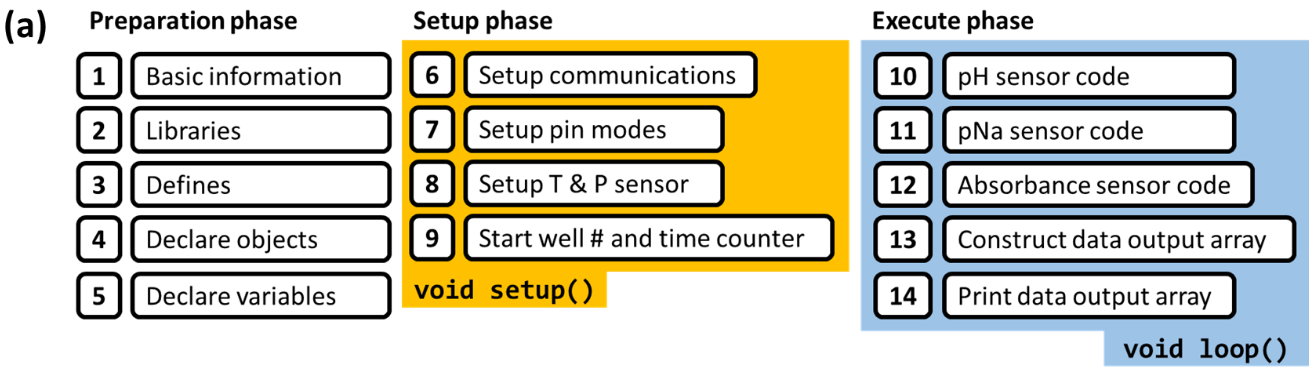

(b)

```
 // Read INA OCP values from cohort 01 electrode 1 ~ 16 indexed 0 ~
15
  if (wellNum < 16) {
    pickChannel(mcp1PHNA, wellNum, COHORT01_S0, COHORT01_S1,
COHORT01_S2, COHORT01_S3);
    delay(5);

    tempPh = adc1.readADC_SingleEnded(0);
    tempVolt = adc1.computeVolts(tempPh);
    phOut[wellNum] = tempVolt;

  }

  else { // Read INA OCP values from cohort 02 electrode 17 ~ 27
indexed 16 ~ 26
    pickChannel(mcp1PHNA, wellNum, COHORT02_S0, COHORT02_S1,
COHORT02_S2, COHORT02_S3);
    delay(5);

    tempPh = adc1.readADC_SingleEnded(1);
    tempVolt = adc1.computeVolts(tempPh);
    phOut[wellNum] = tempVolt;
}
```

(c)

```
for (uint8_t i = 0; i < 84; i++) {
     if (i < 27) {  // pH sensor output index 0 ~ 26
       sensorOut[i] = phOut[i];
     }

     else {
       switch (i) {
           case 27 ... 53:  // Na sensor output index 27 ~ 53
               sensorOut[i] = naOut[i-27];
               break;
           case 54 ... 80: // abs sensor output index 54 ~ 80
               sensorOut[i] = absOut[i-54];
               break;
           case 81: // bmp280 temp sensor output index 81
               sensorOut[i] = tempOut;
               break;
           case 82: // bmp280 pressure sensor output index 82
               sensorOut[i] = pOut;
               break;
           case 83: // lm35 sensor output index 83
               sensorOut[i] = lmTemp;
               break;
         }
       }
     }
```

Figure S10. Firmware structure and code snippets. (a) FW phases; preparation, setup, and execution. (b) Sensor readout code. (c) Output data list construction code.

The 27 electrodes, each positioned within a distinct well, are categorized into two subsets: the initial subset encompasses electrodes 0 through 15 (wells' 1 through 16), and the subsequent subset contains electrodes 16 through 26 (wells 17 through 27). The well number currently being assessed (denoted as the variable wellNum) is ascertained through a conditional if-else statement, which then informs the selection of the multiplexer (MUX) channel via the pickChannel function. Subsequently, a delay of 5 milliseconds is instituted to ensure the MUX has adequately transitioned between channels. Following this, the readADC_SingleEnded() function from the Adafruit_ADS1X15.h library is employed to ascertain the raw data from the ADS1115 ADC, which is then transformed into voltage readings by the computeVolts() function within the same library. The resultant voltage is stored in the phOut array at the index corresponding to wellNum. The coding for the pNa sensor mirrors that of the pH sensor, with the absorbance sensor's code differing only in its division into three groups: wells indexed 0 to 8, 9 to 17, and 18 to 26. The voltage outputs for the pH, pNa, and absorbance sensors are each recorded in separate arrays, named phOut, naOut, and absOut, respectively.

After capturing and allocating the measurements to their respective arrays, the task then shifts to compiling the sensorOut list, which will be used for data output, as illustrated in the code excerpt presented in Figure S10c. This sensorOut list, which extends to 84 elements, amalgamates the data from the phOut, naOut, absOut, alongside additional variables. Subsequent to the assembly of sensorOut, a time stamp—expressed in seconds—is affixed at the forefront, preceding the zeroth index. This augmented list, now complete with the time variable, is then output to the serial port for display and logging purposes.

2.2. Output test

A serial output test was performed on the PCB stack to verify the correct output of measurement values by the OBPC. The Arduino Nano was interfaced with a PC via USB, and the PCB stack received a stable +5 V power supply to ensure adequate voltage and current. Data, consisting of an 85-element list, was transmitted from the Arduino Nano to the PC serially every second. The data, captured as a com-ma-separated value (.csv) file on the PC, was then manually processed with a plotting software. The output, as illustrated in Figure S11a, included one element of time in seconds, 27 elements each for pH and pNa sensor outputs in volts (Figure S11a shows raw ADC values but this was later corrected), 27 elements for absorbance sensor output in volts, followed by two elements for temperature in degrees Celsius and one element for pressure in atmospheres. These outputs were graphically represented in a 2D plot and a 3D waterfall plot, as shown in Figures S11b and S11c, respectively, confirming the operational functionality of the OBPC and its capacity to produce the intended measurement plots through this established procedure.

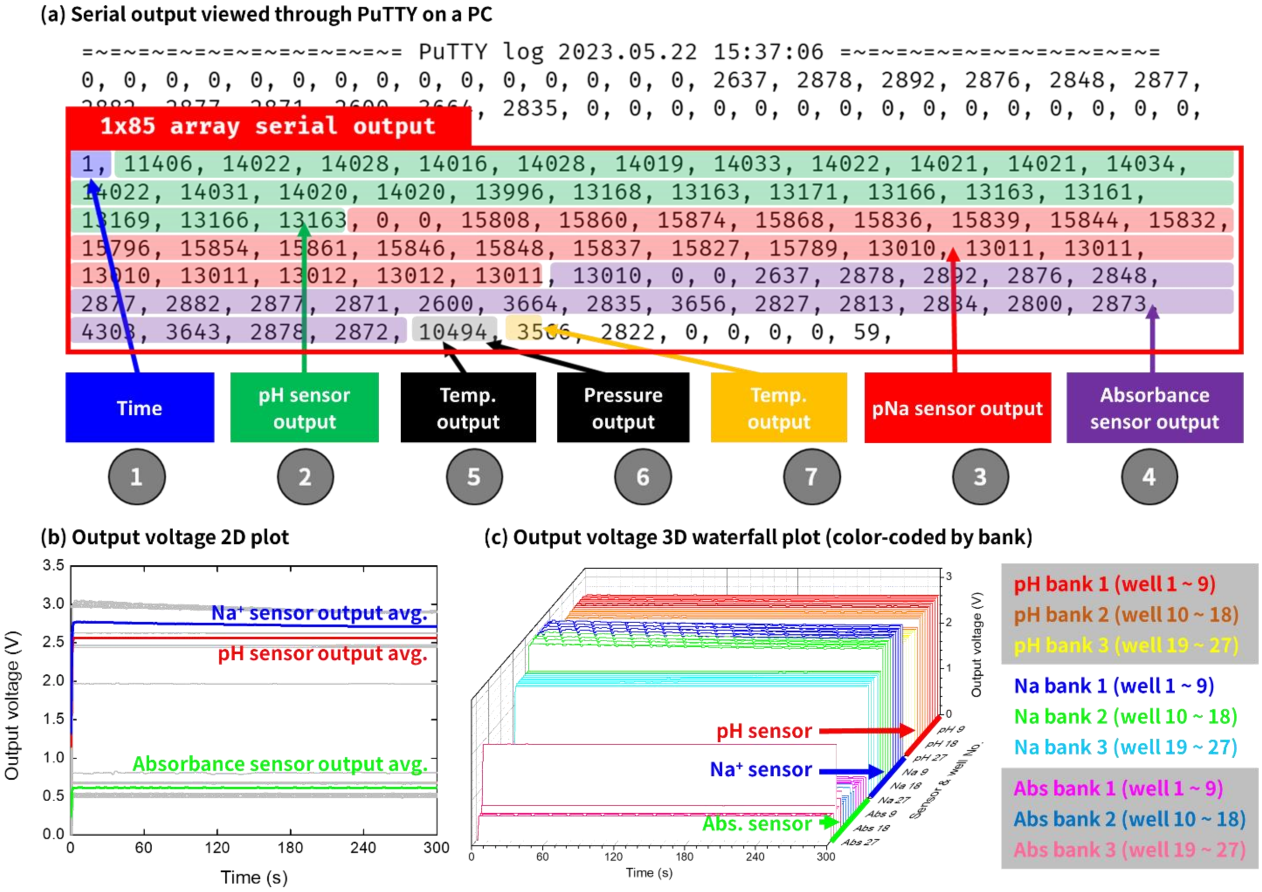

Figure S11. OBPC functional test output. (a) Screenshot of serial monitor showing OBPC output list. (b) 2D and (c) 3D waterfall output voltage plots of all 85 elements from output list.

3. Development model assembly and integration

First, as depicted in Figure S12, the check and solenoid valves are inserted into their designated places within the main mount. Subsequently, the valve bracket is positioned and securely attached to the main mount using M3 nuts and bolts, ensuring the valves are firmly fixed, a process illustrated in Figure S12c. Following this, the payload computer PCBs are methodically arranged atop the main mount, supported by M3 hex stands. The assembly begins with the securing of the first two PCB layers, the aux 1 board and the detector board. Atop this, the fluidic card, along with its 3D printed mount, is placed over the detector board. The final phase involves stacking the remaining two layers, the source board and the aux 2 board, above the fluidic card, as shown in Figure S12e.

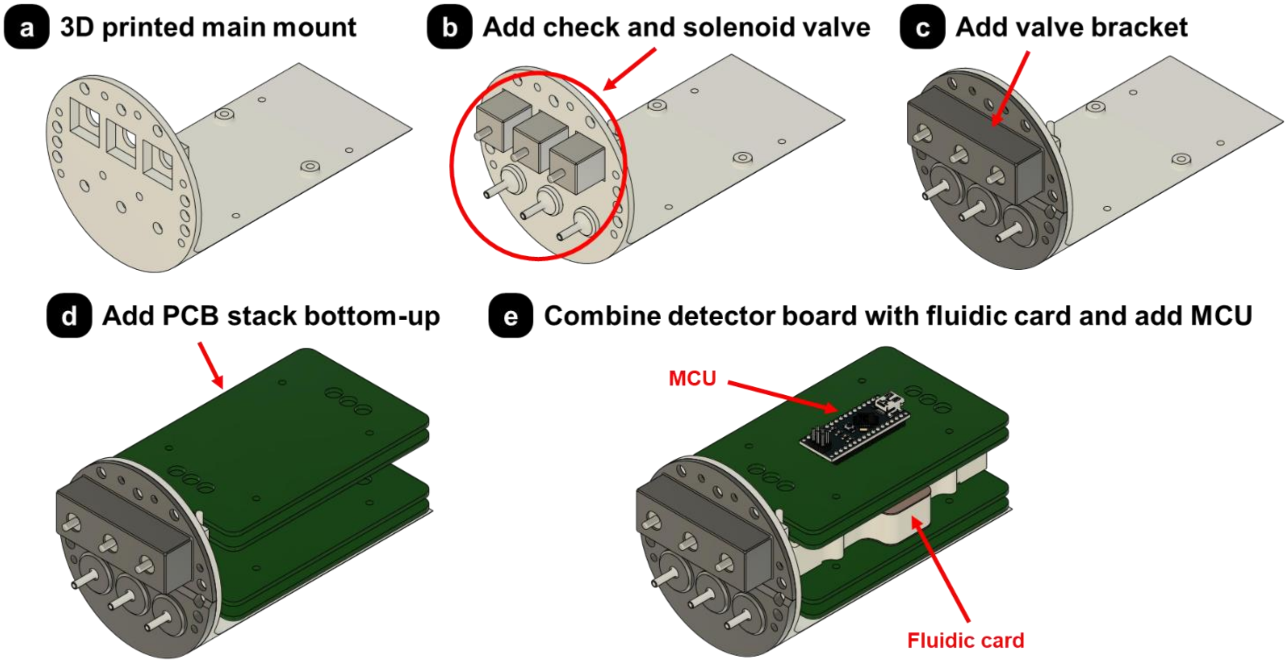

Figure S12. Assembly process of fluidic card, electrode layer, and OBPC. (a) 3D printed main mount. (b) Check and solenoid valve added. (c) Valve bracket added to fasten valves in place. (d) Add PCBs starting from layer 1 to 4 in that order. (e) Add fluidic card with its mount into the stack and finally add the MCU at the top of the PCB stack.

4. Electrochemical Ag/AgCl RE stability across time and pH levels

Figure S13 in the supplementary material thoroughly presents the stability and functionality of the Ag/AgCl reference electrode. Initially, its stability was assessed, with the output of its open circuit potential (OCP) relative to a commercial Ag/AgCl RE over 1000 seconds depicted in Figure S13a. The custom Ag/AgCl pseudo-reference electrode (RE) exhibited reliable performance, sustaining a −13.4 ± 0.9 mV potential (average ± standard deviation) in comparison to a conventional commercial Ag/AgCl RE for a minimum of 15 minutes. This suggests the capability of the developed Ag/AgCl pseudo-RE as an effective substitute for commercial Ag/AgCl REs, capable of maintaining a consistent potential over a certain duration without significant fluctuations.

Furthermore, the electrode's stability across different pH levels, ranging from 6.0 to 4.0, is illustrated in Figure S13b. The resultant OCP demonstrated no marked increase or decrease with pH changes, maintaining a stable average potential of 2379.4 mV. This indicates that the Ag/AgCl RE preserved a stable potential across various pH conditions.

In summary, the experimental findings regarding the potential stability of the Ag/AgCl RE over time and across different pH levels affirm its suitability as an electrochemical reference electrode.

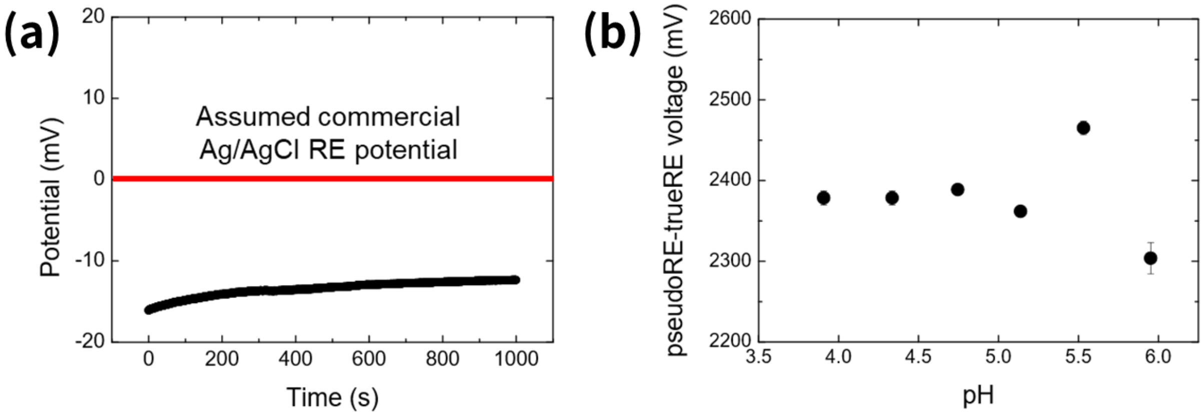

Figure S13. Electrochemical Ag/AgCl RE stability check. OCP data on (a) Ag/AgCl pseudo-RE vs. commercial Ag/AgCl true-RE in 3.5M KCl for 15 min and (b) custom Ag/AgCl pseudo-RE vs. commercial Ag/AgCl true-RE.
